# Multimodal Heartbeat and Compression Optical Coherence Elastography for Mapping Corneal Biomechanics

**DOI:** 10.3389/fmed.2022.833597

**Published:** 2022-04-05

**Authors:** Achuth Nair, Manmohan Singh, Salavat R. Aglyamov, Kirill V. Larin

**Affiliations:** ^1^Biomedical Engineering, University of Houston, Houston TX, United States; ^2^Mechanical Engineering, University of Houston, Houston TX, United States; ^3^Molecular Physiology and Biophysics, Baylor College of Medicine, Houston, TX, United States

**Keywords:** optical coherence elastography (OCE), optical coherence tomography (OCT), biomechanics, cornea, elasticity

## Abstract

The biomechanical properties of the cornea have a profound influence on the health, structural integrity, and function of the eye. Understanding these properties may be critical for diagnosis and identifying disease pathogenesis. This work demonstrates how two different elastography techniques can be combined for a multimodal approach to measuring corneal biomechanical properties. Heartbeat optical coherence elastography (Hb-OCE) and compression OCE were performed simultaneously to measure the stiffness of the cornea in an *in vivo* rabbit model. Measurements were further performed after collagen crosslinking to demonstrate how the combined technique can be used to measure changes in corneal stiffness and map mechanical contrast. The results of this work further suggest that measurements from Hb-OCE and compression OCE are comparable, meaning that Hb-OCE and compression OCE may be used interchangeably despite distinct differences in both techniques.

## Introduction

It is well-established that assessing the mechanical properties of the cornea can help diagnose ocular pathologies or evaluate therapeutic intervention (1, 2). Clinical tools for this assessment include the Corvis ST and the Ocular Response Analyzer (ORA) (3). These tools analyze the corneal response to a large amplitude air puff to assess tissue mechanical properties. However, the large bulk motion and the non-physiological deformation of the cornea results in misleading measurements of mechanical properties (4). As such, alternative tools that can perform a more robust assessment of corneal biomechanical properties are needed.

Elastography has been demonstrably effective for *in vivo* assessment of corneal mechanical properties. Ultrasound elastography has been previously performed in humans using ultrasound surface wave elastography (USWE) and ocular pulse elastography (OPE) (5, 6). However, ultrasonic shear wave techniques typically suffer from poor contrast and a low resolution that can limit their effectiveness for imaging the small, thin ocular tissues. OPE improves on shear wave methods particularly in terms of displacement resolution and sensitivity due to the use of radiofrequency data and interpolation. Furthermore, OPE has been used to successfully distinguish stiffness between untreated and corneal collagen crosslinked human corneas, and represents a significant step forward in approaching the displacement sensitivity of optical imaging techniques (7, 8). Alternatively, Brillouin microscopy has been used to measure the mechanical properties of the cornea *in vivo* (9). However, this technique has long imaging times, and the relationship between the Brillouin modulus and Young’s modulus is still under investigation.

Optical coherence elastography presents another alternative for evaluating biomechanical properties of the cornea *in vivo*. Various techniques, including wave-based OCE, have been performed successfully in humans (10, 11). Compression OCE has been demonstrated in humans as well and has been effective in quantifying mechanical contrast between corneas in healthy subjects and patients with keratoconus (12). In addition to these human studies, both dynamic and quasi-static OCE has been performed in animal studies *in vivo* (13–15). Additionally, passive OCE, a method that involves the use of physiological processes to induce displacement, has also been utilized to study the mechanical properties of the cornea *in vivo* (16, 17). However, OCE has technical challenges as well. Limitations in OCT imaging depth and signal sensitivity rolloff can restrict measurements to the central region of the cornea. Traditional OCT systems also have comparably slower acquisitions speeds compared to other elastography methods, such as ultrasound, which makes real time assessment of elasticity challenging. However, recent advances in OCE using high speed OCT systems seek to overcome some of these obstacles (18, 19).

In this work, we demonstrate a multimodal OCE technique to map the mechanical properties of the cornea that combines a passive technique, heartbeat OCE (Hb-OCE) (17, 20), with an active technique, compression OCE (15, 21). Previously, multimodal optical elastography has only been performed using a combined Brillouin microscopy and OCE system to evaluate the mechanical properties of the crystalline lens (22). However, both techniques were performed separately, and the results were combined for analysis. Here, we demonstrate synchronous multimodal optical elastography for the first time, where both Hb-OCE and compression OCE were used to measure corneal mechanical properties simultaneously. Both methods are quasi-static semi-quantitative (comparible within a region of interest acquired in one measurement) techniques subject to a slow-rate displacement. However, with compression OCE, that displacement comes from a mechanical actuator. In Hb-OCE, the displacement comes in the form of the ocular pulse (23). Here, we demonstrate how a multimodal technique might be used *in vivo* and investigate how these two distinct OCE techniques compare to one another.

## Materials and Methods

Measurements were performed in adult male Dutch-Belted rabbits (*n* = 3). Animals were anesthetized with an initial intramuscular dose of 40 mg/kg ketamine and 5 mg/kg xylazine by a veterinarian. IOP measurements were acquired with a handheld rebound tonometer (Tonovet, Icare Finland Oy, Vantaa, Finland). Animals were placed in a lateral recumbent position in a custom-made rest with a heating pad to ensure that the animals remained at a comfortable temperature throughout the experiments. Vital signs were monitored by a veterinarian, and a maintenance dose of 20 mg/kg ketamine was injected as needed intramuscularly. A three-axis stage was used for orientating the animal under the OCT sample arm. During the procedure, the rabbits were unable to blink, so a solution of 1X phosphate-buffered saline (PBS) was applied topically at regular intervals to maintain corneal hydration. All animal procedures were approved by the University of Houston Institutional Animal Care and Use Committee.

**FIGURE 1:**
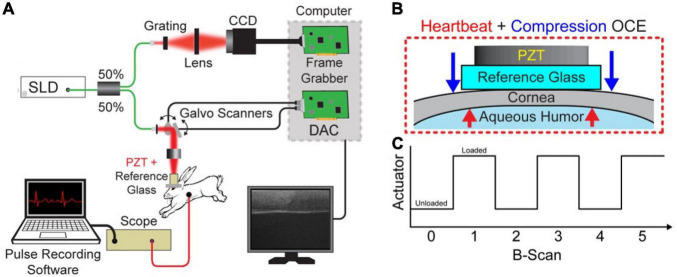
**(A)** Schematic of the combined Hb+Compression OCE system. **(B)** Zoomed in view of the interface between the reference glass and cornea with visualization of the forces acting on the cornea. The blue arrow indicates the compression force applied by the piezoelectric transducer (PZT), and the red arrows illustrate the force applied by the hearbeat-induced ocular pulse. **(C)** PZT actuation as a function of image acquisition, where each B-scan took 16 ms. Hb-OCE data was acquired between two images of the same state (i.e., B-scan 0 versus B-scan 2), and compression OCE measurements were acquired between images of opposite states (i.e., B-scan 0 versus B-scan 1).

The acquisition was performed with a spectral-domain OCT system with 840-nm central wavelength, 49-nm bandwidth, 6-μm axial resolution, and 8-μm lateral resolution operating at a 62.5-kHz line rate. B-M-mode scans of 1000 A-lines were acquired over a ∼2.5 mm region across the apex of the cornea. A hollow cylindrical piezoelectric actuator (HPSt 150/14–10/12 Piezomechanik GmbH, Munich, Germany) mounted to a reference glass was aligned with the OCT sample arm in the common path configuration. Common-path imaging combined with phase-sensitive measurements enabled sub-nanometer displacement sensitivity (21, 24, 25). A small drop of oil was applied to the cornea for lubrication, and the OCT sample arm was lowered onto the cornea until the tissue was applanated within the 2.5 mm field of view. The actuator was synchronized with the OCT frame trigger such that one image was acquired when the tissue was uncompressed, and the next image was acquired when the tissue was compressed (26). As such, compression occurred at the B-scan rate of ∼62 Hz. This low frequency excitation ensures that that there was a minimal effect of viscosity on measurements. Cardiac activity was monitored through the imaging procedure for Hb-OCE measurements using a pressure transducer (PowerLab, ADInstruments, Dunedin, New Zealand) that was placed on the rabbit chest. The heartbeat data was later temporally coregistered with the OCE data by synchronizing the pressure measurements with the time stamps of the OCT images. Figure 1 below shows the schematic of the combined Hb-Compression OCE system, including forces acting upon the cornea and the image acquisition protocol in terms of actuator loading. Compression OCE analysis was performed by comparing images between the compressed and uncompressed states (i.e., B-scan 0 versus B-scan 1 in Figure 1A). On the other hand, Hb-OCE acquisition was performed by comparing OCT images within the same state (i.e., B-scan 0 versus B-scan 2). That leads to an effective B-scan rate of ∼62 Hz for the compression OCE measurements, and ∼31 Hz for Hb-OCE. Since the typical rabbit heart rate is within the range of 3–4 Hz, Hb-OCE measurements were acquired at approximately 10x the heart rate. Images were acquired over 12 s so that multiple cardiac cycles could be acquired, and the corneal strain as a function of time could be detected.

The OCE data were analyzed using a technique described in our previous works (15, 17). Briefly, motion between samples was detected by computing the phase difference between successive frames for analysis using a complex conjugate method (27). Next, a vector averaging method using an isometric 15 μm kernel was used to reduce noise in the phase difference image (28). Phase data, φ, was translated into displacement *d* using the following relationship,


(1) $$d=\frac{\varphi \lambda_{0}}{4\pi n},$$

where, λ_0_ was the central wavelength of the OCT system, and *n* was the refractive index, considered to be 1.376 in the cornea (29). The OCT displacement stability was measured to be less than <0.5 nm at a signal to noise ratio of 65 dB (20). The strain was calculated using a least-squares regression algorithm along every A-line in the displacement image within an isometric window with a size of 75 μm (30). Regions at the periphery of the field of view with poor contact with the reference glass were not included in the analysis. Hb-OCE measurements were temporally filtered at the heart rate measured by the pressure transducer, then mapped and quantified based on the peak strain averaged over 12 cardiac cycles. On the other hand, compression OCE images were calculated based on mean strain within the same period. Thus, the analysis was performed on data acquired at the same time.

To compare the capabilities of both techniques for assessing changes in corneal stiffness and mapping mechanical heterogeneity, UV-A/riboflavin corneal collagen crosslinking (CXL) was performed on the corneas using the standard Dresden protocol following measurement of the untreated cornea (31). Therefore, of the three animals used for measurements, *n* = 6 eyes were used for untreated measurements, and *n* = 2 eyes, one eye from each of two animals, were utilized for crosslinked measurements. Data from one virgin cornea was removed due to poor contact with the reference glass. Due to the low sample size of CXL corneas, inter-sample statistical testing was not performed. Changes in stiffness due to CXL were assessed using intra-sample statistical testing, as each image pixel indicates a unique measurement. Briefly, the corneal epithelium was removed, and a solution of 0.1% riboflavin in 20% dextran was topically applied to the surface of the cornea in 5-minute intervals for 60 min. After 30 min of riboflavin instillation, the surface of the cornea was irradiated with UV light (365 nm, 3 mW/cm^2^). Partial CXL was also performed in one cornea by limiting the UV irradiation area to a localized region of the cornea. The raw partial CXL data was also utilized in a previous publication (15).

## Results

**FIGURE 2:**
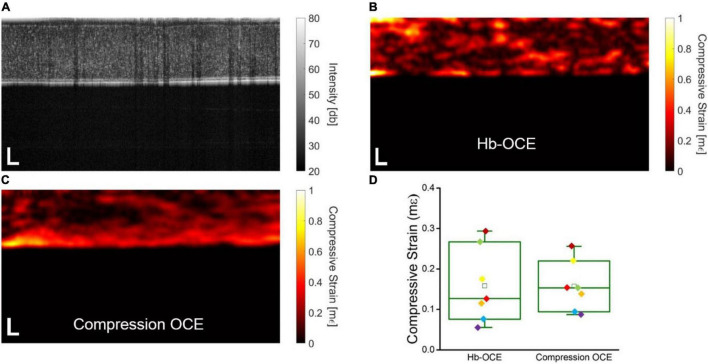
**(A)** OCT intensity B-scan of a representative virgin cornea. Corneal stiffness mapped by **(B)** Hb-OCE and **(C)** compression OCE. **(D)** Box and whisker plot comparing measurements performed by Hb-OCE and compression OCE (*n* = 7). The box shows median and upper and lower quartiles, and the whiskers represent the minimum and maximum data points. Scale bars indicate 100 μm.

Figure 2A shows the OCT intensity B-scan of a representative virgin cornea. The compressive strain measured by Hb-OCE and compression OCE are shown in Figures 2B,C, respectively. Both corneas show a slight degree of spatial heterogeneity, as expected. Figure 2D shows a direct comparison between Hb-OCE and compression OCE measurements (*n* = 5 virgin and *n* = 2 CXL corneas). The inter-sample mean compressive strain measured by Hb-OCE was 0.16 ± 0.9 mε, and 0.16 ± 0.6 mε measured by compression OCE. The measurements from Hb-OCE and compression OCE were statistically insignificant by a paired *t*-test (*p* = 0.96).

**FIGURE 3:**
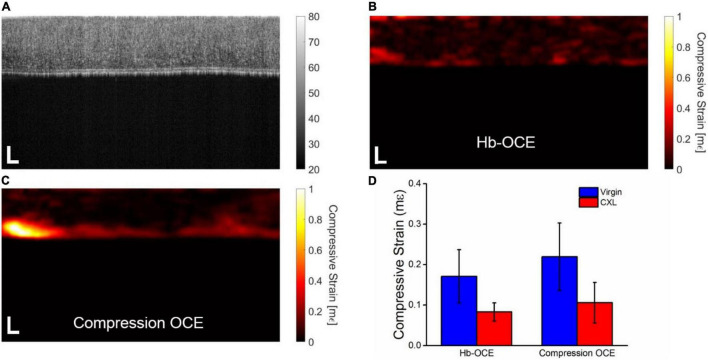
**(A)** OCT intensity B-scan of the cornea in Figure 2 that has been crosslinked. Corneal stiffness mapped by **(B)** Hb-OCE and **(C)** compression OCE **(D)** Mean and standard deviation of virgin and crosslinked corneas measured by Hb-OCE and compression OCE. Scale bars are 100 μm.

Figure 3A shows the OCT intensity B-scan of the cornea from Figure 2A that has undergone CXL. Note the increase in scattering after CXL as compared to Figure 2A as well as the decrease in the overall thickness of the tissue, consistent with our previous results with CXL (32). Figures 3B,C illustrate the compressive strain in the cornea measured by Hb-OCE and compression OCE, again showing an overall spatial homogeneity in the cornea. Strain is noticeably lower with both measurements techniques when compared to the cornea before the CXL treatment in Figure 2. Because there were only two samples in the crosslinking group, inter-sample statistical testing would not provide meaningful information. As such, Figure 3D shows the corneal strain measured by Hb-OCE and compression OCE of the same cornea before and after CXL. The mean compressive strain measured by Hb-OCE in the virgin cornea was 0.17 ± 0.06 mε and 0.07 ± 0.02 mε in the CXL cornea. The mean compressive strain measured by compression OCE was 0.22 ± 0.08 mε in the virgin cornea and 0.10 ± 0.06 mε in the CXL cornea. A paired *t*-test showed a statistically insignificant difference between Hb-OCE measurements and compression OCE measurements in the virgin corneas (*p* = 0.79), and a statistically insignificant difference between both OCE techniques in the CXL cornea (*p* = 0.87). However, both Hb-OCE and compression-OCE showed a statistically significant difference between untreated and crosslinked tissues by a paired *t*-test (*p* < 0.01 and *p* = 0.038, respectively). Both methods also demonstrated a greater than 60% increase in the stiffness of both corneas after crosslinking was performed, which is within expectations (15).

**FIGURE 4:**
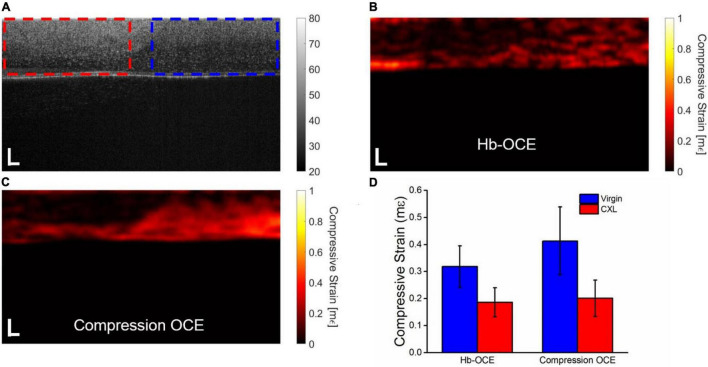
**(A)** OCT intensity B-scan of a partially crosslinked cornea. The dotted boxes indicate the (red) crosslinked and (blue) virgin regions, respectively. Corneal stiffness mapped by **(B)** Hb-OCE and **(C)** compression OCE. **(D)** Mean and standard deviation of virgin and crosslinked regions measured by Hb-OCE and compression OCE. Scale bars are 100 μm.

Figure 4 illustrates the results of both Hb-OCE and compression OCE in a partially crosslinked rabbit cornea. Figure 4A shows the OCT intensity B-scan, where the dotted regions are the calculation windows of the (red) CXL and (blue) virgin regions. Figures 4B,C illustrate the compressive strain map obtained by Hb-OCE and compression OCE, respectively. Both maps show a distinct difference in corneal strain between the CXL and virgin regions. Figure 4D plots the mean compressive strain measured by Hb-OCE and compression OCE in the virgin and CXL regions. Hb-OCE measured a mean compressive strain of 0.32 ± 0.07 mε in the virgin region and a mean compressive strain of 0.18 ± 0.05 mε in the CXL region. Compression OCE measured a mean compressive strain of 0.41 ± 0.13 mε in the virgin region and 0.20 ± 0.07 mε in the CXL region. A paired *t*-test showed a statistically insignificant difference between Hb-OCE and compression OCE measurements in both the virgin (*p* = 0.58) and CXL (*p* = 0.08) regions. However, between the untreated and CXL regions, both Hb-OCE (*p* < 0.01) and compression OCE (*p* = 0.007) showed a statistically significant difference. Hb-OCE showed a 43% increase in stiffness after CXL, and compression OCE showed a 56% increase in stiffness after CXL.

## Discussion

In this work, we performed a pilot study to demonstrate the first use of multimodal OCE using a simultaneous acquisition to our knowledge. Hb-OCE and compression OCE have been previously used to measure the biomechanical properties of the cornea *in vivo*, and though both methods can be useful to provide semi-quantitative information regarding corneal biomechanical properties, the techniques have not been compared to one another directly despite their similarities. Hb-OCE and compression OCE are distinguished by two primary factors. First, displacement in Hb-OCE is passively induced by the ocular pulse, whereas in compression OCE, displacement is actively induced using a mechanical actuator. As a result, in this work, mechanical actuation in compression OCE occurs ∼14x faster than heart rate, which is beneficial for patient comfort. Second, in Hb-OCE, the displacement occurs from within the aqueous humor below the cornea, and in compression OCE, the displacement occurs from above the tissue. Finally, the stress and subsequent displacement induced by the mechanical actuator is far more easily controlled and repeatable due to its electro-mechanical nature as compared to the ocular pulse. Despite these differences, based on the results of Figure 2, mean strains from Hb-OCE and compression OCE are statistically insignificant, suggesting that both measurement techniques may effectively provide equivalent information despite their differences. Presumably, this was because the ocular pulse stress and the PZT stress were very similar, resulting in similar strains. This finding suggests that Hb-OCE may be a suitable passive replacement for compression OCE for mapping corneal strains and vice versa. The current study has a limited sample size, with multiple measurements being performed on three animals. While the data here does suggest certain trends, it is important to note that future studies involving a larger sample size are required to obtain more conclusive results.

The results described in Figure 3 further support that Hb-OCE and compression OCE may provide comparable measurements. Hb-OCE and compression OCE were used to compare the change in corneal stiffness after CXL. While both techniques showed a significant difference between virgin and crosslinked corneas, there was not a significant difference between measurements performed by either technique. We can see this occurrence in Figure 4 as well in a partially crosslinked cornea. While there is a statistically significant difference between both regions measured by both techniques, the difference between techniques for a particular region is still insignificant. Furthermore, while compression OCE has been used to map mechanical contrast in corneas *in vivo* (15), Figure 4 shows the first use of Hb-OCE for the task.

However, our results also suggest certain limitations. The results of our work show significant variance in compressive strain measured by compression OCE, which is highlighted in Figures 3, 4. This variance is likely due to axial variation in stiffness between the anterior and posterior segments of the cornea (15). Furthermore, variances may be due to noise introduced by additive numerical errors from each step of the calculation, which will be addressed in future improvements to the strain calculation algorithm. It should be noted that the increased corneal strain around the endothelium shown in the compression OCE strain maps is likely due to edge artifacts related to the strain calculation and was not used for statistical analysis. Compression OCE has been previously used to demonstrate differences between these two regions (15); however, it is less clear from these results if Hb-OCE can distinguish these regions. On the other hand, Hb-OCE shows significantly less variance from the mean compressive strain. This is largely in part due to the slightly reduced displacement of Hb-OCE (0.05 ± 0.03 μm) compared to compression OCE (0.07 ± 0.04 μm), which may not reach all the way through the cornea. In fact, while mechanical resolution and sensitivity have been investigated in compression OCE (33, 34), neither has been evaluated for *in vivo* quasi-static OCE. Further investigation will be needed to clarify.

Our study is also subject to measurement limitations as well. Direct applanation of the cornea, as shown here, may not be comfortable for a patient. However, Goldmann applanation tonometry (GAT) is typically performed with a topical anesthetic, although GAT was well tolerated even without anesthetic (35). Furthermore, there is precedence for applanating the cornea for OCE (12, 36). Additionally, applanation is known to alter the IOP of the eye globe, and due to the relationship between IOP, ocular pulse amplitude, and the Hb-OCE measurements, this change in IOP must be accounted for. In our measurements of Hb-OCE, we allow the eye globe enough time to stabilize so that transient changes in IOP due to the applanation process have limited effects on the Hb-OCE measurements. Finally, this study has been limited to the 2D mapping of axial strains, however various algorithms have been developed to assess lateral and axial strains using compression OCE (37, 38). Future work will investigate how these algorithms can be implemented into Hb-OCE measurements. Beyond 2D mapping of corneal stiffness, 3D mapping would be even more valuable. Mapping corneal strain in 3D using compression OCE may require considerable acquisition times with the current setup, and 3D Hb-OCE is still under investigation. However, ultra-fast OCE techniques may be able to limit or even eliminate the increase in imaging time going from 2D to 3D measurements (18, 19). Furthermore, while this study focuses on the measurement of axial strain, further developing compression OCE and Hb-OCE with 3D applications is an avenue of our future work.

Here, we performed semi-quantitative mapping of corneal biomechanical properties using a multimodal Hb-OCE and compression OCE technique. However, because strain is a semi-quantitative metric, comparisons between samples based on strain are innately limited. To translate these measurements to truly quantitative results, we need to quantify the stress acting upon the cornea due to either compression or the ocular pulse in addition to the strain. One way to perform this assessment is by quantifying the IOP *in vivo*, which may be performed using dynamic contour tonometry (6). Measuring the change in IOP as a response to the compression OCE measurement or recording the change in IOP due to the ocular pulse could be useful in quantifying corneal stress and translating strain into a quantitative biomechanical parameter (15). Another option is the use of a compliant stress sensor, which has been demonstrated in previous compression OCE applications (39). A stress sensor would be especially useful for mapping heterogeneity in stress and could provide an even more accurate measurement of stiffness using Hb-OCE and compression OCE with the added benefit of increased mechanical contrast. Furthermore, it should be noted that the corneal strain measured by Hb-OCE could be heavily influenced by the ocular pulse. Further analysis that accounts for the ocular pulse, or the stress on the cornea, would allow for more accurate comparison between these two types of measurements.

## Conclusion

This work demonstrates multimodal Hb-OCE and compression OCE for the first time *in vivo*. Both techniques were used to measure the change in corneal stiffness after CXL and showed a similar increase in stiffness. Furthermore, both Hb-OCE and compression OCE were used to map mechanical contrast in a sample that had been partially crosslinked. Most importantly, our results showed that despite distinctly different methods of action for both techniques, the strain quantified by Hb-OCE and compression OCE were largely similar, suggesting that both techniques may be used interchangeably to measure corneal stiffness *in vivo*.

## Data Availability Statement

The raw data supporting the conclusions of this article will be made available by the authors, without undue reservation.

## Ethics Statement

The animal study was reviewed and approved by UH IACUC.

## Author Contributions

All authors developed the concept, reviewed the data, and reviewed the manuscript.

## Conflict of Interest

The authors declare that the research was conducted in the absence of any commercial or financial relationships that could be construed as a potential conflict of interest.

## Publisher’s Note

All claims expressed in this article are solely those of the authors and do not necessarily represent those of their affiliated organizations, or those of the publisher, the editors and the reviewers. Any product that may be evaluated in this article, or claim that may be made by its manufacturer, is not guaranteed or endorsed by the publisher.
